# Analysis of the efficacy and mechanism of action of Buzhong Yiqi Decoction on gastric cancer using meta-analysis and network pharmacology

**DOI:** 10.1097/MD.0000000000049076

**Published:** 2026-05-29

**Authors:** Ruiping Yang, Yonggui Wu, Chunhui Tao, Xiaojing Lin, Ruixue Jiang

**Affiliations:** a The First Affiliated Hospital of Guangzhou University of Chinese Medicine, Guangzhou, Guangdong, China; b Basic Medical Sciences College, Hubei University of Chinese Medicine, Wuhan, Hubei, China; c Clinical School of Traditional Chinese Medicine, Hubei University of Chinese Medicine, Wuhan, Hubei, China.

**Keywords:** Buzhong Yiqi Decoction, gastric cancer, meta-analysis, molecular docking, network pharmacology

## Abstract

**Background:**

To explore the efficacy and mechanism of Buzhong Yiqi Decoction (BZYQD) in the treatment of gastric cancer (GC) using meta-analysis and network pharmacology.

**Methods:**

We searched PubMed, Embase, Cochrane Library, Chinese BioMedical Literature Database, China National Knowledge Infrastructure, China Science and Technology Journal (CQVIP), and Wanfang databases for randomized controlled trials that evaluated BZYQD therapy for GC. The outcomes were clinical efficacy, 2-year survival rate, Karnofsky performance status score, and incidence of adverse reactions. We performed a meta-analysis using RevMan 5.3 software. Then, the potential active ingredients and targets of BZYQD were screened through the Traditional Chinese Medicine Systems Pharmacology. GC-related targets were screened through the GeneCards database. The “active ingredients-targets” network was constructed by Cytoscape 3.8.0 software, and the protein–protein interaction network was constructed using the STRING database. Gene Ontology and Kyoto Encyclopedia of Genes and Genomes enrichment analyses of the effective targets of BZYQD in GC were performed through the Metascape platform. Finally, molecular docking technology was used to identify the affinity and activity between core targets and active compounds.

**Results:**

A total of 15 randomized controlled trials were included in the literature. This literature included a total of 1176 patients, of whom 588 were in the treatment group and 588 were in the control group. In terms of efficacy indicators and incidence of adverse reactions, the treatment group was better than the control group. There was a statistical difference (*P* < .05). A total of 150 active ingredients of BZYQD and 136 targets acting on GC were identified. A total of 2430 Gene Ontology terms and 172 Kyoto Encyclopedia of Genes and Genomes enrichment entries were obtained using enrichment analysis, involving apoptosis and the phosphoinositide 3-kinase/protein kinase B signaling pathway. The high affinity and activity of the core targets and active compounds were verified through molecular docking.

**Conclusion:**

This study demonstrated the efficacy and safety of BZYQD in the treatment of GC, and the related molecular mechanisms were initially revealed.

## 1. Introduction

Gastric cancer (GC), the most prevalent gastrointestinal malignant tumor worldwide, has an ever-increasing prevalence. According to statistics released in 2024, both the morbidity and mortality of GC rank fifth among global malignant tumors, and GC results in 968,350 newly diagnosed cases and 659,853 deaths in 2022.^[1]^ At present, the pathogenesis of GC has not been thoroughly elucidated. The existing knowledge indicates that bacteria (such as *Helicobacter pylori*), smoking, and inherited mutations of specific genes (such as the *CDH1* gene) may be prominent risk factors for GC.^[2]^ Currently, surgical resection remains the standard therapeutic method for GC patients, but unfortunately, the majority of GC patients have already progressed to the middle and advanced stages when they are diagnosed. Moreover, chemotherapy for GC treatment is often accompanied by various toxic side effects such as gastrointestinal discomfort, myelosuppression, and liver dysfunction.^[3]^ Against this backdrop, it is of great significance to explore alternative or adjuvant treatment methods with few side effects and favorable curative efficacy for GC patients.

Buzhong Yiqi Decoction (BZYQD) is derived from the “Spleen and Stomach Theory” written by Li Dongyuan, a famous doctor in the Jin Dynasty. BZYQD is composed of 8 kinds of herbs: Astragalus membranaceus (“Huangqi” in Chinese), Rhizoma Atractylodis Macrocephalae (“Baizhu” in Chinese), Radix Ginseng (“Renshen” in Chinese), Radix Glycyrrhizae (“Gancao” in Chinese), Pericarpium Citri Reticulatae (“Chenpi” in Chinese), Radix Bupleuri (“Chaihu” in Chinese), Radix Angelicae Sinensis (“Danggui” in Chinese), and Rhizoma Cimicifugae (“Shengma” in Chinese). In recent years, many studies have shown that BZYQD helps to improve the clinical efficacy of GC, reduce toxic side effects, improve quality of life, and prolong the survival of patients. BZYQD can restore the damaged immune function and increase the lymphocyte infiltration of gastric tumors, showing notable antitumor effects.^[4,5]^ Therefore, the clinical efficacy and mechanism of BZYQD on GC deserve further validation.

**Figure 1.**
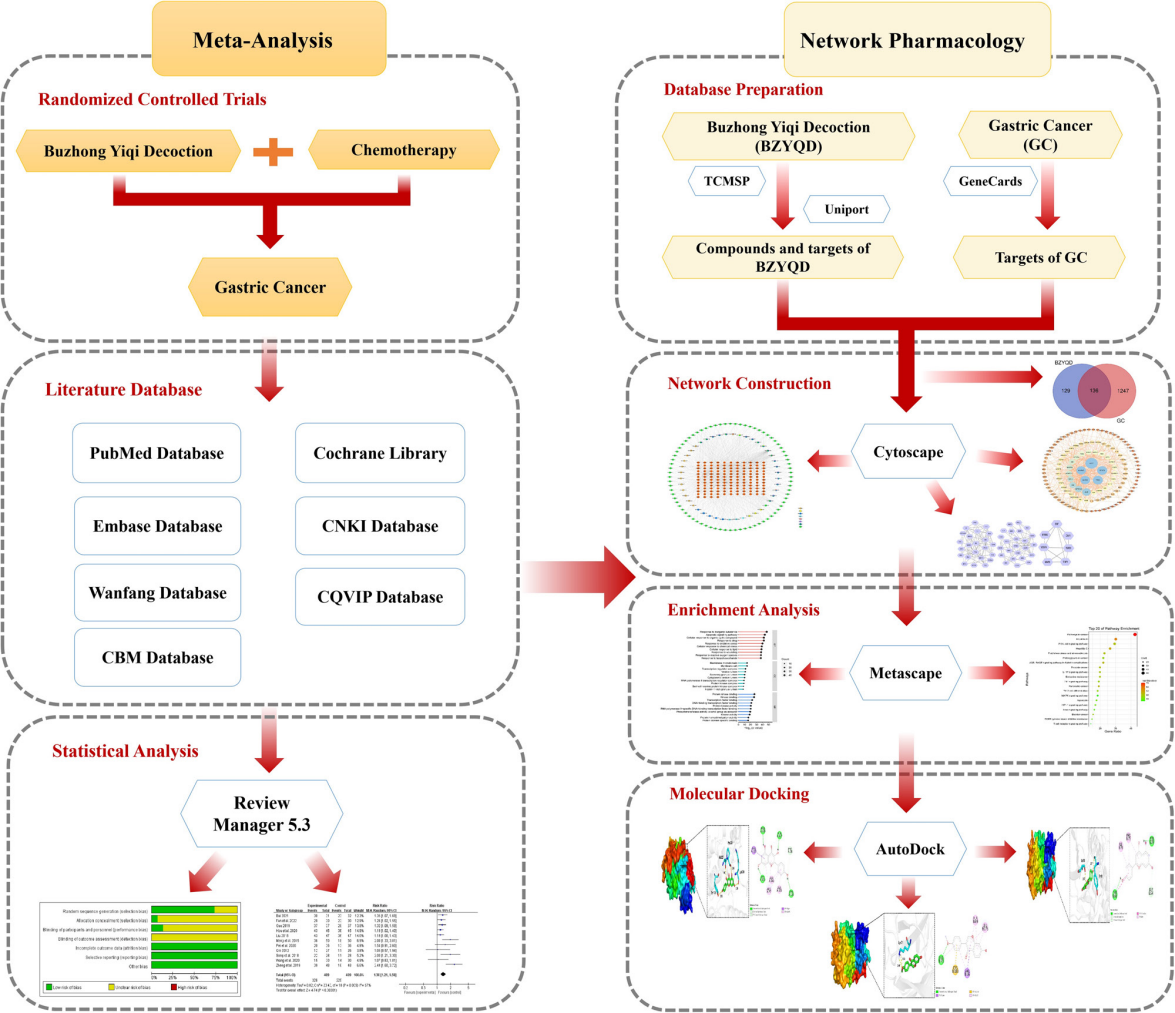
The flowchart of the analysis procedures of the study. BZYQD = Buzhong Yiqi Decoction, GC = gastric cancer, TCMSP = Traditional Chinese Medicine Systems Pharmacology.

Meta-analysis, as the main analysis method to provide a decision-making basis for evidence-based medicine, can comprehensively analyze multiple research results qualitatively and quantitatively; meta-analysis results are important evidence to prove the efficacy and rationality of a drug or compound.^[6]^ On the other hand, network pharmacology, an emerging discipline based on systems biology and computer technology, has been extensively applied in the traditional Chinese medicine compound research field with its functionality of predicting drug effective ingredients, targets, and toxic side effects.^[7]^ In the present study, meta-analysis and network pharmacology were combined to evaluate the efficacy and safety of BZYQD in GC treatment and to predict its active ingredients and related targets and signaling pathways, aiming to provide novel insights for the evidence-based medical research of BZYQD on GC. The detailed flowchart of the present study is shown in Figure 1.

## 2. Materials and methods

### 2.1. Search strategy

PubMed, Embase, Cochrane Library, Chinese BioMedical Literature, China National Knowledge Infrastructure, China Science and Technology Journal (CQVIP), and Wanfang databases were retrieved from the database establishment time to May 1, 2022. The terms (“gastric cancer”/“stomach cancer,” “Buzhong Yiqi decoction,” and “randomized controlled trial”/“RCT”) were retrieved in titles and abstracts of literature. There was no limitation of language, date, or file type of literature to reduce the risk of publication bias.

### 2.2. Inclusion and exclusion criteria

The inclusion criteria were randomized controlled trials (RCTs); GC diagnosed by pathology; the treatment group was treated with BZYQD combined with chemotherapy, and the control group was only treated with chemotherapy alone; and outcome indicators were clinical efficacy, 2-year survival rate, Karnofsky performance status (KPS) score, and incidence of adverse reactions. The exclusion criteria were no diagnosis by pathological examination; no relevant outcome data; non-RCT literature (such as reviews and animal experiments); and combined use of physical therapy methods (such as acupuncture and massage).

### 2.3. Data extraction and quality assessment

Data (the first author, year of publication, sample size, age, intervention measures, course of treatment, and outcome indicators) were extracted from the included literature by 2 independent researchers. Subsequently, the quality of the included literature was assessed by 2 independent researchers using the Cochrane Collaboration’s tool.^[8]^ When disagreements were encountered, a decision was made through discussion or by a third researcher.

### 2.4. Data integration and statistical analysis

Meta-analysis was performed using RevMan 5.3 software (The Nordic Cochrane Centre, The Cochrane Collaboration). The relative risk (RR) and the mean difference were used as the effect index for the enumeration data and measurement data, respectively, both of which were expressed with a 95% confidence interval (CI). The fixed-effects model was used for analysis if there was little or no heterogeneity among the included studies (*P* > .1, *I*^2^ ≤ 50%); otherwise, the random-effects model was used for analysis (*P* ≤ .1, *I*^2^ > 50%).

### 2.5. Screening of active ingredients and targets of BZYQD

Following the selection criteria of oral bioavailability ≥ 30% and drug-likeness ≥ 0.18,^[9]^ the active ingredients of 8 herbs in BZYQD were searched through the Traditional Chinese Medicine Systems Pharmacology (https://tcmsp‐e.com/tcmsp.php).^[10]^ After that, the targets corresponding to each active ingredient were retrieved. Finally, the collected target names were converted into the corresponding gene symbols using the UniProt database (https://www.uniprot.org/).^[11]^

### 2.6. Screening of GC-related targets and construction of “Active Ingredients-Targets” network

By searching the keywords “gastric cancer,” the GC-related targets were collected from the GeneCards database (https://www.genecards.org/).^[12]^ The targets corresponding to the active ingredients of BZYQD and GC-related targets were intersected, and the intersection of the 2 was the predicted action target of BZYQD on GC. The “active ingredients-targets” network about BZYQD in GC treatment was constructed using Cytoscape 3.8.0 (Cytoscape Consortium, http://www.cytoscape.org/) and then the network was analyzed through the Network Analyzer plug-in.

### 2.7. Construction of protein–protein interaction (PPI) network and cluster analysis

The intersected targets were imported into the STRING database (https://www.string-db.org/) for PPI analysis.^[13]^ We used the Cytoscape 3.8.0 software to build a PPI network and conducted a topological analysis with the CytoNCA plug-in, and then the core targets were screened based on the degree value. Some target proteins with the same or similar characteristics and functions in cell biological activities were regarded as a cluster. Proteins in the same cluster are generally thought to play a synergistic role in disease progression.^[14]^ Therefore, the MCODE plug-in of Cytoscape 3.8.0 software was used to perform cluster analysis of target proteins in bioinformatics networks.

### 2.8. Gene Ontology (GO) and Kyoto Encyclopedia of Genes and Genomes (KEGG) enrichment analysis

The Metascape platform (www.metascape.org/)^[15]^ was used to perform GO and KEGG enrichment analysis on the intersection targets of BZYQD and GC. The results of enrichment analysis were reserved and finally visualized using the R software (version 3.6.3; R Foundation for Statistical Computing).

### 2.9. Molecular docking simulations

The top 5 key components in degree value in the “active ingredients-targets” network were molecularly docked with the top 5 core targets in the PPI network to verify the affinity and activity between components and targets. The structures of key compounds were obtained from the PubChem database (https://pubchem.ncbi.nlm.nih.gov),^[16]^ and the structures of the core targets were obtained from the Research Collaboratory for Structural Bioinformatics Protein Data Bank (RCSB PDB, https://www.rcsb.org/).^[17]^ The molecular docking simulation was performed using AutoDock (The Scripps Research Institute) software and finally visualized using PyMOL (Schrödinger, LLC) software.

## 3. Results

### 3.1. The efficacy of BZYQD on GC

#### 3.1.1. Literature retrieval results and basic features

**Figure 2.**
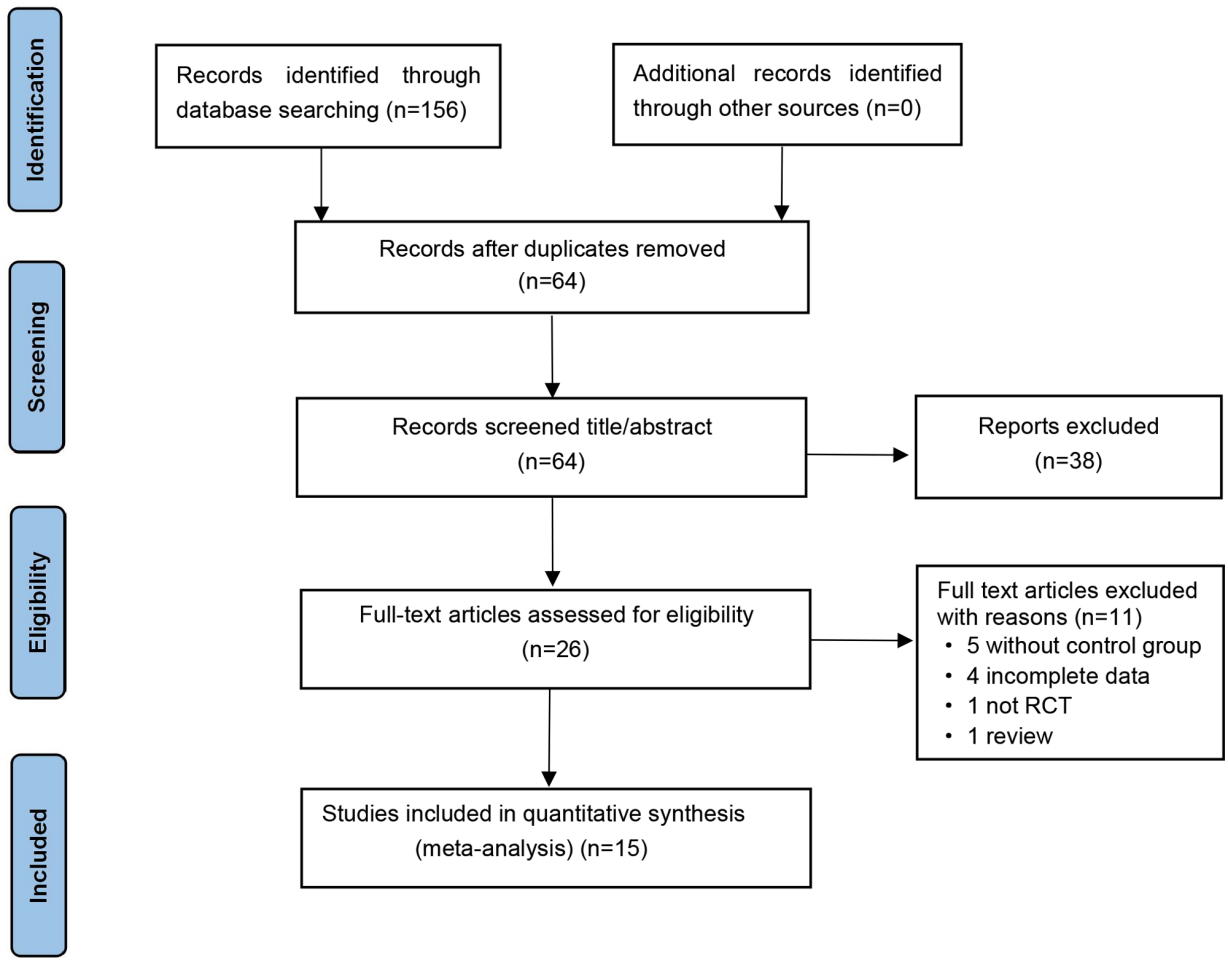
Flowchart of the literature retrieval process. RCT = randomized controlled trial.

**Figure 3.**
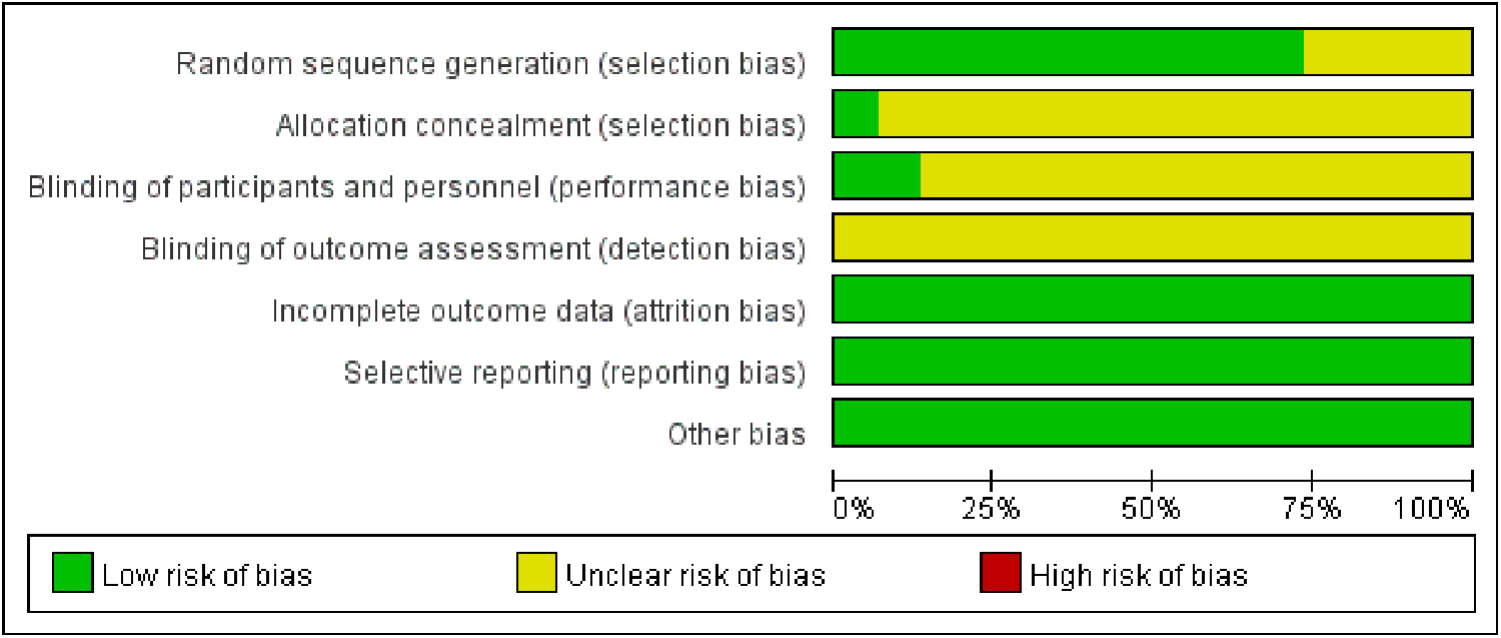
Risk of bias graph for all the included studies.

A total of 15 RCTs were included, and 1176 patients (588 in the treatment group and 588 in the control group) were involved. The literature screening process is shown in Figure 2. The basic features and risk of bias of included studies are shown in Table 1 and Figure 3.

**Table 1:** Basic characteristics of the included studies.

Author, yr	Sample size (T/C)	Age (yr) (T/C)	Treatment group	Control group	Duration	Outcomes
Hou et al, 2020^[18]^	45/45	66.07 ± 8.54/66.38 ± 3.75	BZYQD + chemotherapy	XELOX chemotherapy	6 wk	①②⑤⑥
Fan et al, 2022^[19]^	30/30	70.55 ± 2.04/70.56 ± 2.03	BZYQD + chemotherapy	XELOX chemotherapy	9 wk	①③
Wang et al, 2020^[20]^	30/30	50.18 ± 3.82/49.33 ± 5.09	BZYQD + chemotherapy	XELOX chemotherapy	2 mo/6 wk	①③④⑥
Bai, 2021^[21]^	31/32	49.50 ± 3.10/50.00 ± 3.12	BZYQD + chemotherapy	XELOX chemotherapy	2 mo/6 wk	①
Meng et al, 2015^[22]^	50/50	51.2 ± 7.8	BZYQD + chemotherapy	FOLFOX chemotherapy	3 mo	①②④⑤
Zheng et al, 2019^[23]^	48/48	58.26 ± 7.48/57.81 ± 8.14	BZYQD + chemotherapy	FOLFOX chemotherapy	3 mo	①
Gao, 2019^[24]^	37/37	55.26 ± 10.27/55.21 ± 10.21	BZYQD + chemotherapy	FOLFOX chemotherapy	4 wk	①④
Song et al, 2018^[25]^	28/28	55.76 ± 10.48/55.87 ± 10.65	BZYQD + chemotherapy	FOLFOX chemotherapy	3 mo	①②④⑤
Liu, 2018^[26]^	47/47	51.31 ± 7.31/52.37 ± 7.54	BZYQD + chemotherapy	FOLFOX chemotherapy	3 mo	①④⑤
Qin, 2012^[27]^	27/26	59.04 ± 8.72/57.73 ± 10.72	BZYQD + chemotherapy	SOX chemotherapy	6 wk	①③④⑥
Pei et al, 2020^[28]^	36/36	56.37 ± 10.41/54.72 ± 12.68	BZYQD + chemotherapy	TCF chemotherapy	8 wk	①⑤
Sun et al, 2021^[29]^	40/40	55.11 ± 5.22/55.32 ± 6.17	BZYQD + chemotherapy	XELOX chemotherapy	3 mo	②③⑤⑥
Chen, 2021^[30]^	45/45	66.45 ± 3.68/66.38 ± 3.75	BZYQD + chemotherapy	XELOX chemotherapy	6 wk	③④⑥
Xu et al, 2016^[31]^	48/48	54.90 ± 5.6/54.80 ± 5.9	BZYQD + chemotherapy	FOLFOX chemotherapy	8 wk	④⑤⑥
Xie et al, 2020^[32]^	46/46	48.52 ± 3.44/48.56 ± 3.41	BZYQD + chemotherapy	TPF chemotherapy	6 wk	④

① clinical efficacy; ② 2-year survival rate; ③ KPS score; ④ gastrointestinal symptoms; ⑤ myelosuppression; ⑥ liver dysfunction.

BZYQD = Buzhong Yiqi Decoction, C = control group, FOLFOX = 5-fluorouracil + calcium folinate + oxaliplatin, KPS = Karnofsky performance status, SOX = oxaliplatin + gimeracil and oteracil potassium capsules, T = treatment group, TCF = Taxol + cisplatin + 5-fluorouracil, TPF = docetaxel + cisplatin + 5-fluorouracil, XELOX = capecitabine + oxaliplatin.

#### 3.1.2. Clinical efficacy

**Figure 4.**
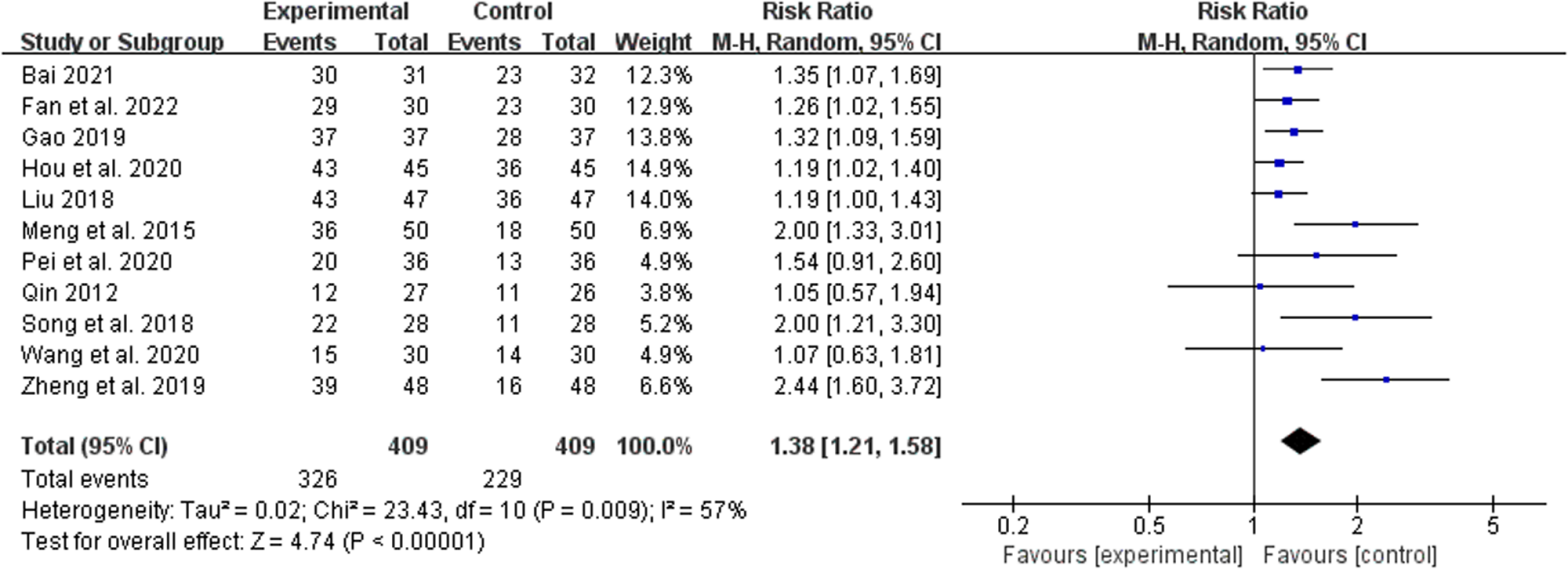
Forest plot of clinical efficacy of BZYQD plus chemotherapy versus chemotherapy therapy alone. BZYQD = Buzhong Yiqi Decoction, CI = confidence interval.

A total of 11 studies^[18–28]^ reported the clinical efficacy. The random-effects model was used for analysis due to statistical heterogeneity among the results of each study (*P* = .009, *I*^2^ = 57%). Meta-analysis results revealed a statistically significant difference between the treatment group and the control group (RR = 1.38, 95% CI [1.121, 1.58]; *P *< .00001), which indicated that the treatment group had a remarkably elevated clinical efficacy rate relative to the control group (Fig. 4).

#### 3.1.3. Two-year survival rate

**Figure 5.**
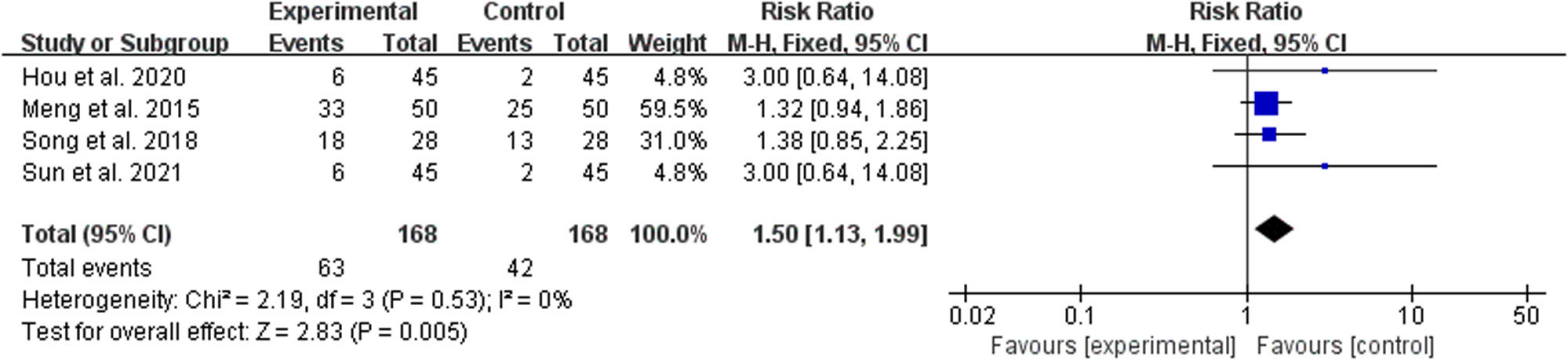
Forest plot of 2-year survival rate of BZYQD plus chemotherapy versus chemotherapy therapy alone. BZYQD = Buzhong Yiqi Decoction, CI = confidence interval.

The 2-year survival rate was reported in 4 studies.^[18,22,25,29]^ No statistical heterogeneity was found among the results of the studies (*P* = .53, *I*^2^ = 0%), and the fixed-effects model was used for analysis. According to the meta-analysis results of the 2-year survival rate, a statistically significant difference between the treatment group and the control group was noted (RR = 1.50, 95% CI [1.13, 1.99]; *P* = .005), which indicated that the treatment group had a noticeably higher 2-year survival rate than the control group (Fig. 5).

#### 3.1.4. KPS score

**Figure 6.**
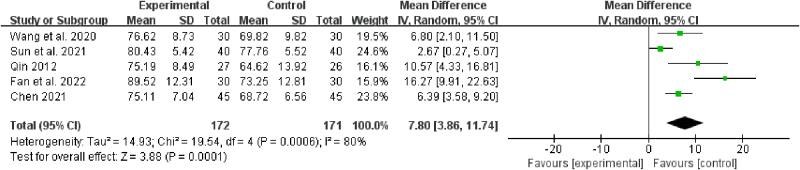
Forest plot of the KPS score of BZYQD plus chemotherapy versus chemotherapy therapy alone. BZYQD = Buzhong Yiqi Decoction, CI = confidence interval, KPS = Karnofsky performance status, SD = standard deviation.

The KPS score was reported in 5 studies.^[19,20,27,29,30]^ There was statistical heterogeneity among the results of each study (*P* = .00006, *I*^2^ = 80%), and the random-effects model was used for analysis. As shown by meta-analysis results, the statistically significant difference between the 2 groups (mean difference = 7.80, 95% CI [3.86, 11.74]; *P* = .0001) suggested a remarkably higher quality of life of patients in the treatment group than that in the control group (Fig. 6).

#### 3.1.5. Incidence of adverse reactions

**Figure 7.**
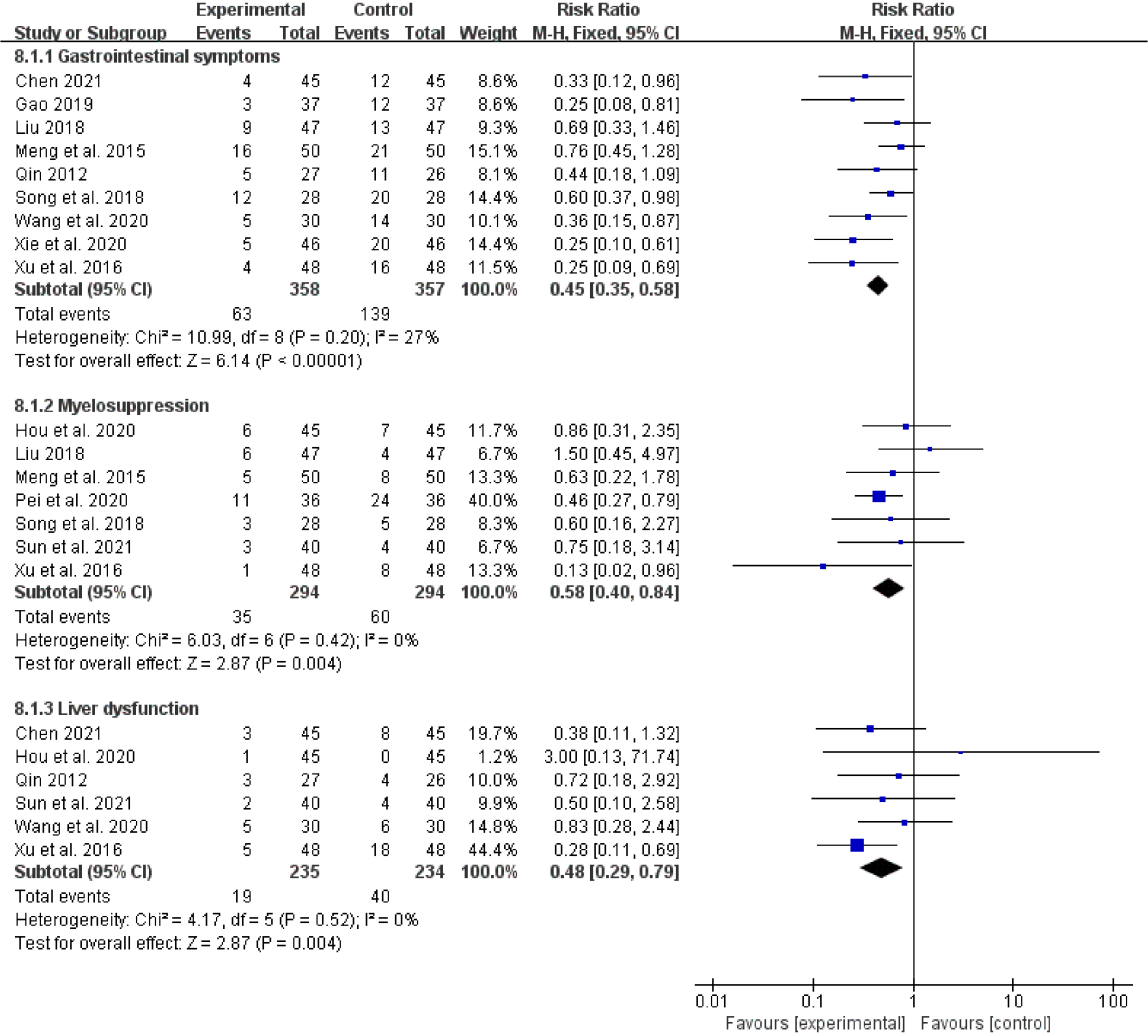
Forest plot of incidence of adverse reactions of BZYQD plus chemotherapy versus chemotherapy therapy alone. BZYQD = Buzhong Yiqi Decoction, CI = confidence interval.

Gastrointestinal symptoms (9 studies,^[20,22,24–27,30–32]^ *I*^2^ = 27%), myelosuppression (7 studies,^[18,22,25,26,28,29,31]^ *I*^2^ = 0%), and liver dysfunction (6 studies,^[18,20,27,29–31]^ *I*^2^ = 0%) were the primary adverse reactions reported in the included studies. No statistical heterogeneity among the study results was observed, and the fixed-effects model was used for analysis. As revealed by the meta-analysis results, there were statistically significant differences in the incidence of adverse reactions (*P* < .00001, *P* = .004, and *P *= .004, respectively) between the 2 groups, indicating that the treatment group had a notably reduced incidence of adverse reactions relative to the control group (Fig. 7).

### 3.2. Mechanism of BZYQD on GC

#### 3.2.1. Active ingredients and targets of BZYQD

Through the Traditional Chinese Medicine Systems Pharmacology database, 20 components of Astragalus membranaceus, 7 components of Rhizoma Atractylodis Macrocephalae, 20 components of Radix Bupleuri, 5 components of Pericarpium Citri Reticulatae, 2 components of Radix Angelicae Sinensis, 92 components of Radix Glycyrrhizae, 22 components of Radix Ginseng, and 17 components of Rhizoma Cimicifugae were retrieved. After excluding the duplicate values, 150 active ingredients of BZYQD were obtained. Finally, a total of 265 targets corresponding to the active ingredients were identified.

#### 3.2.2. GC-related targets and the “Active Ingredients-Targets” network

**Figure 8.**
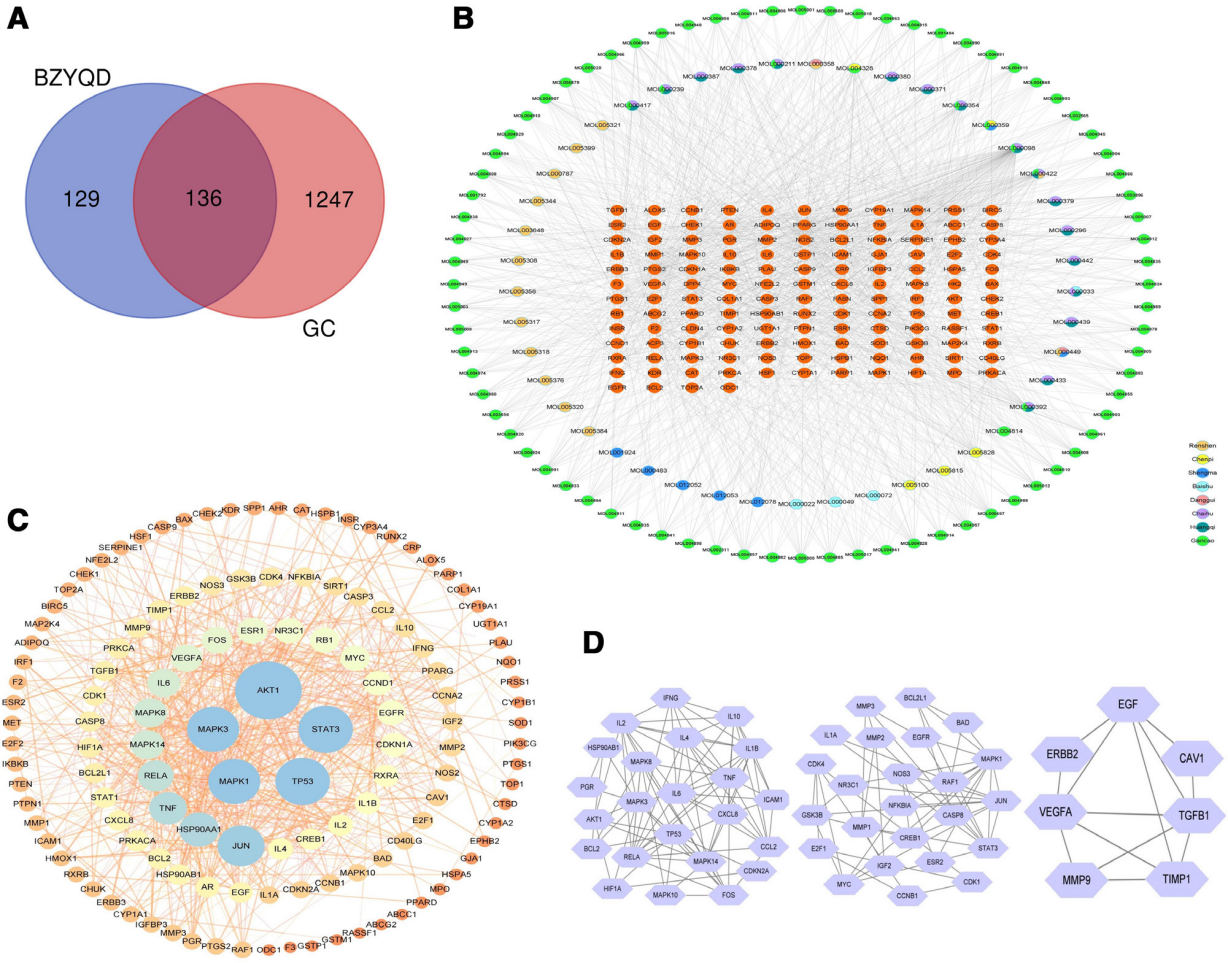
Screening of compounds and targets for BZYQD in the treatment of GC. (A) Venn diagram of intersection between BZYQD (blue) and GC (red) targets. (B) “active ingredients-targets” network. The nodes in the outer circle represent all the active ingredients of BZYQD in the treatment of GC; the dark orange nodes in the middle represent the targets of BZYQD acting on the GC. (C) PPI network. The sizes and colors of the nodes and edges are illustrated from big to small and blue to orange in descending order of degree value. (D) MCODE clustering in the PPI network. BZYQD = Buzhong Yiqi Decoction, GC = gastric cancer, PPI = protein–protein interaction.

A total of 12,136 GC targets were obtained from the GeneCards database, and 1383 GC targets were finally obtained after screening (relevance score > 10). The Venn diagram was used to map the targets of BZYQD and the targets of GC (Fig. 8A), and finally, 136 intersected targets were identified. Next, the intersected targets and active ingredients were introduced into Cytoscape 3.8.0 software for constructing the “active ingredients-targets” network (Fig. 8B). The network was analyzed through the Network Analyzer plug-in. The larger the degree value is, the more important the corresponding compound is in the network.^[33]^ The top 5 active ingredients with the degree value in the network were quercetin, kaempferol, nobiletin, naringenin, and formononetin (Table 2).

**Table 2:** Information of the top 5 active ingredients in BZYQD by their degree value.

Molecule ID	Active ingredient	OB (%)	DL	Degree
MOL000098	Quercetin	46.43	0.28	104
MOL000422	Kaempferol	41.88	0.24	58
MOL005828	Nobiletin	61.67	0.52	52
MOL004328	Naringenin	59.29	0.21	48
MOL000392	Formononetin	69.67	0.21	37

BZYQD = Buzhong Yiqi Decoction, DL = drug-likeness, OB = oral bioavailability.

#### 3.2.3. PPI network and cluster analysis

The intersected targets were imported into the STRING database for PPI analysis, and then the PPI network was constructed using Cytoscape 3.8.0 software (Fig. 8C). The CytoNCA plug-in was used for topology analysis. The size and color of the nodes reflect the importance of the degree value. The greater the degree value, the higher the importance of nodes in the network.^[34]^ It was found that the top 5 targets with degree value were AKT serine/threonine kinase 1 (AKT1; degree = 45), signal transducer and activator of transcription 3 (STAT3; degree = 40), tumor protein p53 (TP53; degree = 40), mitogen-activated protein kinase 1 (MAPK1; degree = 39), and mitogen-activated protein kinase 3 (MAPK3; degree = 39). Finally, 3 modules were extracted from the PPI network using the MCODE plug-in (Fig. 8D).

#### 3.2.4. GO and KEGG enrichment analysis

**Figure 9.**
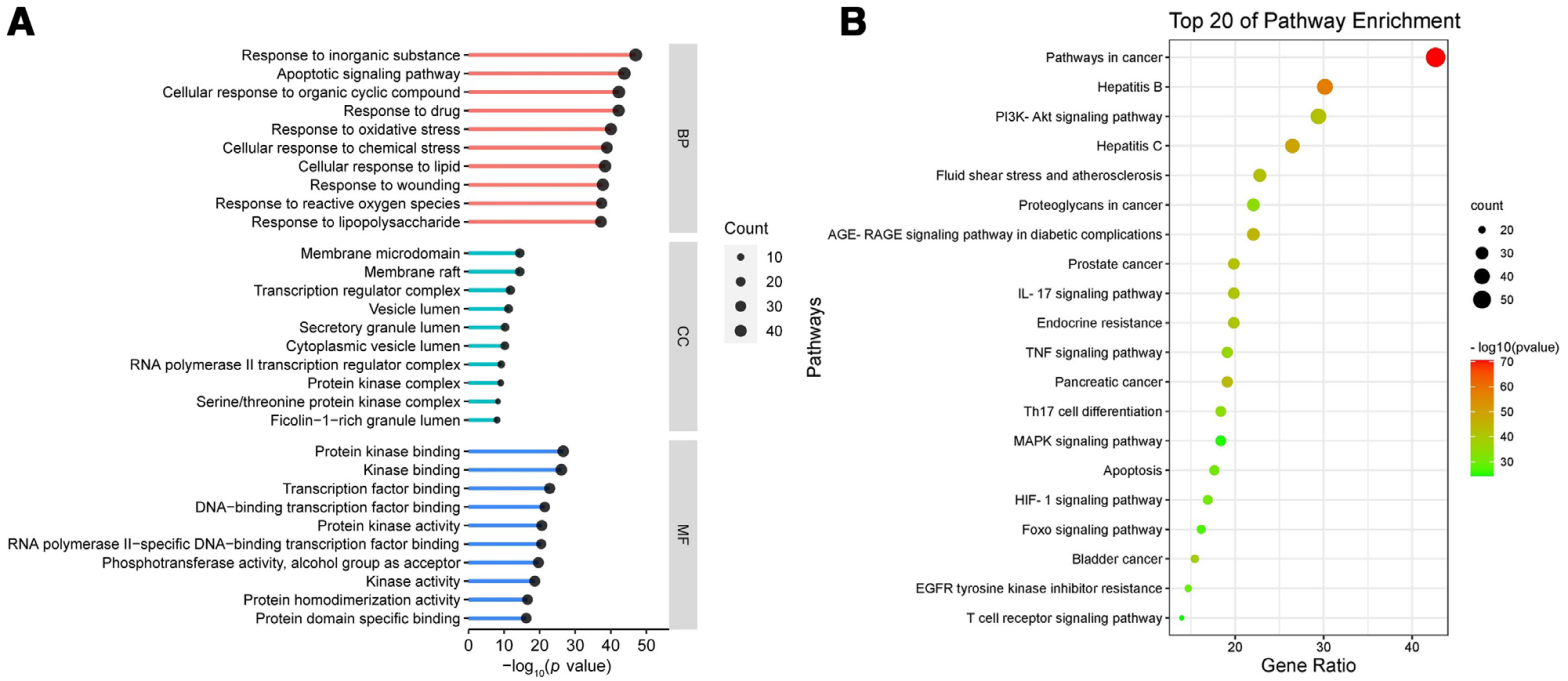
Enrichment analysis of the targets of BZYQD in the treatment of GC. (A) GO enrichment analysis: each bar indicates a GO term on the vertical axis. The bubble in the bar represents the number of genes enriched in each GO term. The larger the bubble, the more genes in the GO term. The length of each bar is determined by the *P*-value. The longer the bar, the smaller the *P*-value. (B) KEGG enrichment analysis: each bubble represents a KEGG pathway on the vertical axis. The size of the bubble represents the number of genes enriched in each KEGG pathway, and the larger the bubble, the more genes in the pathway. The color of each bubble is determined by the *P*-value, and the redder the color of the bubble, the smaller the *P*-value. BP = biological process, BZYQD = Buzhong Yiqi Decoction, CC = cell component, GC = gastric cancer, GO = Gene Ontology, KEGG = Kyoto Encyclopedia of Genes and Genomes, MF = molecular function.

GO and KEGG enrichment analyses were performed on the intersected targets using the Metascape platform. As shown in the GO enrichment analysis results, there were 2208 biological process (BP) entries, 77 cell component entries, and 145 molecular function entries, and the top 10 results of BP, cell component, and molecular function were selected, respectively, according to the *P*-value to plot a bar chart (Fig. 9A). Through KEGG pathway enrichment analysis, 172 signaling pathways were obtained, and the top 20 results were selected according to the *P*-value to make a bubble diagram (Fig. 9B).

#### 3.2.5. Molecular docking verification

**Figure 10.**
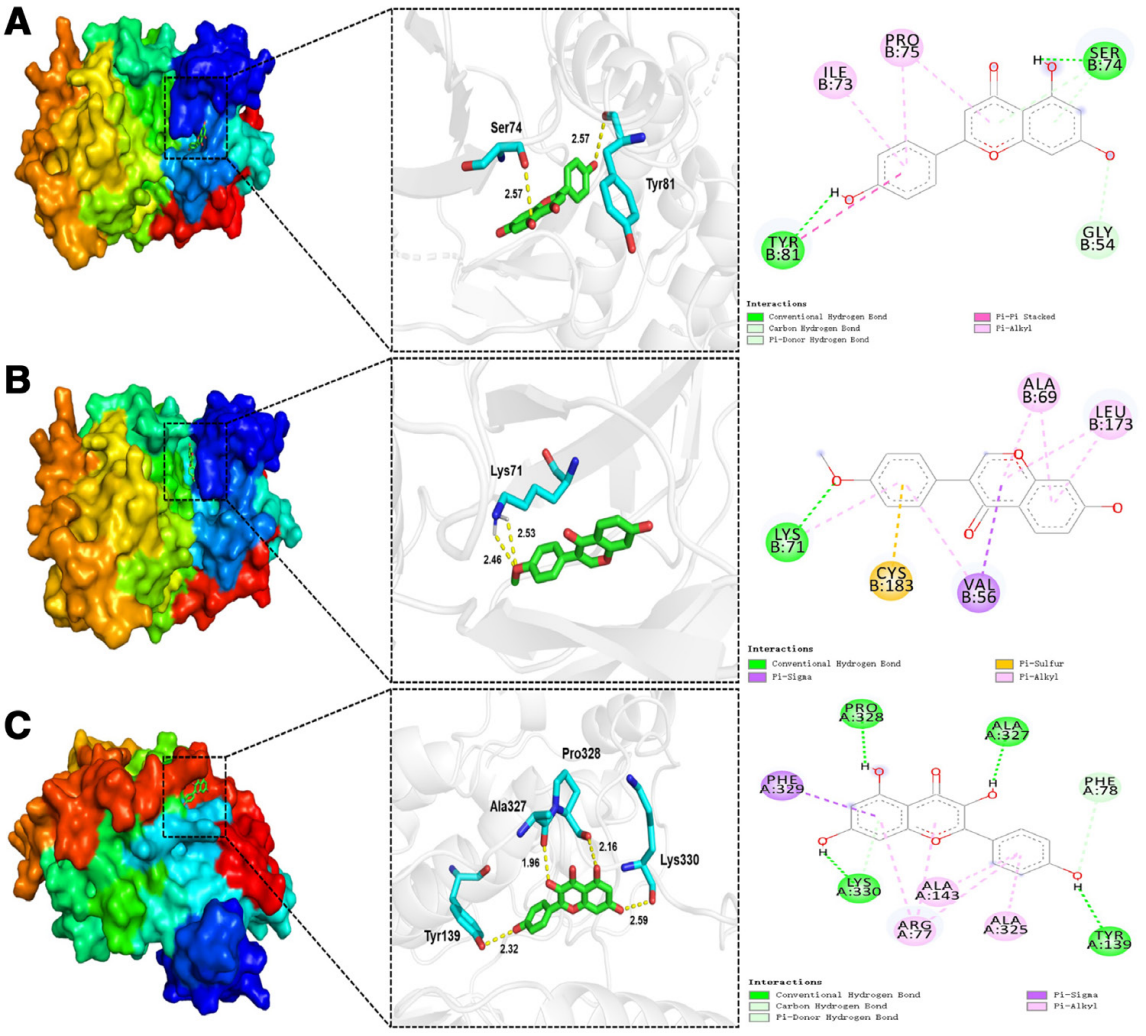
Molecular docking diagram. (A) Naringenin-MAPK3. (B) Formononetin-MAPK3. (C) Kaempferol-MAPK. MAPK3 = mitogen-activated protein kinase 3.

The top 5 key compounds (quercetin, kaempferol, nobiletin, naringenin, formononetin) and the top 5 core targets (AKT1, STAT3, TP53, MAPK1, MAPK3) were selected for molecular docking using AutoDock software (Table 3). The results demonstrated that all the key compounds can bind well with the core targets, with MAPK3-naringenin showing the best binding energy (−8.08 kcal/mol; Fig. 10A). The molecule formed 2 hydrogen bonds with the protein amino acid residues Ser74 and Tyr81 at a distance of 2.57 Å. Moreover, it formed a hydrophobic interaction with Gly54, Ile73, Pro75, and Tyr81. The second and third binding scores were MAPK3-formononetin (−7.97 kcal/mol) and MAPK1-kaempferol (−7.92 kcal/mol), respectively (Fig. 10B and C).

**Table 3:** Docking scores of key compounds and core targets (kcal/mol).

	AKT1 (PDB ID: 5AAR)	STAT3 (PDB ID: 6QHD)	TP53 (PDB ID: 6IU7)	MAPK1 (PDB ID: 6G54)	MAPK3 (PDB ID: 4QTB)
Quercetin	−6.04	−5.57	−5.44	−6.04	−7.20
Kaempferol	−6.43	−5.59	−5.48	−7.92	−7.19
Nobiletin	−5.31	−4.67	−4.46	−4.70	−5.91
Naringenin	−6.45	−5.59	−5.88	−6.88	−8.08
Formononetin	−6.39	−6.64	−6.46	−6.90	−7.97

AKT1 = AKT serine/threonine kinase 1, MAPK1 = mitogen-activated protein kinase 1, MAPK3 = mitogen-activated protein kinase 3, STAT3 = signal transducer and activator of transcription 3, TP53 = tumor protein p53.

#### 3.2.6. External validation

In addition, we also searched the experimental literature on the effects of the key compounds in BZYQD on the core targets of GC. Finally, we collected 9 studies^[35–43]^ (8 in vitro experiments, 1 in vitro + in vivo experiment), as shown in Table 4. The results of these studies show that the key compounds of BZYQD can act on the core targets in multiple ways, thereby intervening in GC progression, which greatly supports the findings of our study.

**Table 4:** Summary of experimental studies of key compounds related to the core targets.

Compounds	Targets	Cell line	In vitro/in vivo	Dose	Duration	Results	References
Quercetin	TP53	Human gastric cancer cell line SGC-7901	In vitro	0, 20, 40, and 60 μM	24, 48, and 72 h	Increasing the expression of tumor suppressor gene TP53, thereby inhibiting the proliferation and migration of gastric cancer cells.	Shao et al^[35]^
Quercetin	AKT	Human gastric cancer cell line MGC-803	In vitro	53 μM	24 h	Reducing the expression of AKT phosphorylation and inducing gastric cancer cells apoptosis via the mitochondria-dependent pathway.	Shen et al^[36]^
Quercetin	STAT3	Human gastric cancer cell line MGC-803	In vitro	20, 40, and 60 μM	48 h	Reducing expression of STAT3 phosphorylation, thereby inhibiting gastric cancer cell proliferation and inducing apoptosis.	Qin et al^[37]^
Kaempferol	AKT	MNK-28 and SGC-7901 and GSE-1 cells	In vitro; in vivo (mice)	0, 60, and 120 μM; 20 mg/kg/d	24, 48, and 72 h; 3 wk	Decreasing the level of AKT phosphorylation inhibiting the proliferation of gastric cancer cells, and inducing apoptosis and cell cycle arrest, but no effect on normal gastric GSE-1 cells.	Song et al^[38]^
Kaempferol	TP53	Human gastric cancer cell line SGC-7901	In vitro	50,100, and 200 μM	24 h	Increasing the expression of TP53, thereby promoting the apoptosis of gastric cancer cells.	Wang et al^[39]^
Nobiletin	STAT3	Human gastric cancer cell line SGC-7901	In vitro	0, 30, 60, and 120 mg/L	24 h	Reducing the expression of STAT3 phosphorylation, inhibiting EMT, thereby suppressing the proliferation and invasion of gastric cancer cells.	Yang et al^[40]^
Naringenin	AKT	Human gastric cancer cell line SGC-7901	In vitro	20, 40, and 80 μM	24, 48, and 72 h	Reducing the expression of Akt phosphorylation, suppressing the proliferation, migration, and invasion of gastric cancer cells, and promoting apoptosis.	Bao et al^[41]^
Naringenin	TP53	Human gastric cancer cell line SGC-7901	In vitro	40 μM	48 h	Increasing the expression of TP53, reducing Akt activation, and inhibiting the proliferation of gastric cancer cells.	Zhang et al^[42]^
Formononetin	AKT	Human gastric cancer cell line MGC-803	In vitro	0, 20, 40, and 80 μM	48 h	Reducing the expression of AKT phosphorylation, inhibiting the proliferation of gastric cancer cells and promoting apoptosis.	Liu et al^[43]^

AKT = protein kinase B, EMT = epithelial-mesenchymal transition, STAT3 = signal transducer and activator of transcription 3, TP53 = tumor protein p53.

## 4. Discussion

Surgery, as the first-choice treatment for GC, has been internationally recognized as the only method for the radical treatment of GC. Postoperative chemotherapy helps to kill residual tumor cells within the body, thereby improving surgical efficacy and controlling the primary lesions.^[44]^ However, postoperative chemotherapy is often accompanied by various toxic side effects, such as nausea, vomiting, myelosuppression, and liver dysfunction. In recent years, BZYQD has been frequently used as an adjuvant method of chemotherapy for the treatment of GC patients in China, which can improve clinical efficacy and greatly reduce adverse reactions.

Therefore, in this study, the effectiveness and safety of BZYQD for GC treatment were first evaluated using an evidence-based analytical approach. A total of 15 RCTs were screened for meta-analysis. After comparing the indicators of BZYQD combined with chemotherapy and chemotherapy alone in the treatment of GC, the results indicated that BZYQD can significantly improve the clinical efficacy, quality of life, and patient survival rate, as well as greatly reduce the incidence of adverse reactions. It is noteworthy that significant statistical heterogeneity was observed in the pooled analyses of “clinical efficacy” and “KPS score” in this study. Although the use of a random-effects model partially mitigated the impact of this heterogeneity, the underlying causes remain to be explored. We speculate that the heterogeneity may primarily stem from 2 aspects: first, the combination chemotherapy regimens adopted by the included studies varied (e.g., XELOX, FOLFOX, SOX, etc), and differences in drug combinations, dosages, and mechanisms of action might have led to inconsistencies in efficacy outcomes; second, the treatment duration differed across trials, which may have variably influenced patients’ long-term functional status (as reflected by KPS score). These factors represent one of the limitations of this study, and future rigorously designed trials with higher homogeneity are warranted for further validation.

Network pharmacology advocates multicomponent therapy, which is consistent with the characteristics of traditional Chinese medicine therapy (multicomponent, multitarget, and multipathway). Through the network pharmacology approach, we found that the key compounds of BZYQD in the treatment of GC may be quercetin, kaempferol, nobiletin, naringenin, and formononetin. Prior studies have shown that quercetin can regulate the phosphoinositide 3-kinase/protein kinase B (PI3K/Akt) pathway by affecting AKT phosphorylation, downregulating AKT kinase, and inhibiting epithelial-mesenchymal transition in GC cells, thereby repressing GC cell migration and invasion.^[45]^ Kaempferol can promote GC cell autophagy, increase the conversion from LC3-I to LC3-II, and downregulate the expression of p62 protein in GC.^[46]^ Nobiletin triggers endoplasmic reticulum stress-mediated apoptosis and GC cell autophagy via the downregulation of the Akt/mechanistic target of rapamycin signaling pathway.^[47]^ Naringenin can inhibit GC cell proliferation, migration, and invasion, and induce apoptosis by inhibiting the phosphorylation of AKT and decreasing the expression of its downstream target molecules.^[41]^ Formononetin can depress the growth and migration of GC cells by regulating the Wnt/β-catenin and AKT/mechanistic target of rapamycin pathways.^[48]^

The results of the PPI analysis showed that the targets of BZYQD related to the treatment of GC may mainly be AKT1, STAT3, TP53, MAPK1, and MAPK3. Akt is a protein kinase that regulates the cell cycle and apoptosis. Activated Akt can interact with several key downstream effectors and phosphorylate them, thereby suppressing cell apoptosis.^[49]^ In the tumor microenvironment, tumor cells can secrete interleukins, which in turn activate the STAT3 signaling pathway, leading to GC cell progression and deterioration.^[50]^ TP53 is commonly accepted as a tumor suppressor gene. TP53 expression is continuously increased during the deterioration of normal gastric mucosa into intestinal metaplasia and then into GC, and TP53 mutation can be frequently observed in GC patients.^[51]^ MAPK is widely implicated in numerous vital BPs such as cell proliferation, differentiation, and apoptosis through phosphorylation of nuclear transcription factors and related enzymes.^[52]^

GO enrichment analysis indicated that BZYQD exerts effects on GC through a variety of pivotal BPs, such as apoptosis, oxidative stress, chemical stress, and lipopolysaccharide reaction. KEGG pathway enrichment analysis exhibited that the main pathways of BZYQD in the treatment of GC included apoptosis, PI3K/Akt signaling pathway, proteoglycan in cancer, interleukin-17 signaling pathway, tumor necrosis factor (TNF) signaling pathway, and hypoxia-inducible factor 1 (HIF-1) signaling pathway. Over the years, the relationship between apoptosis and GC has been extensively investigated by researchers.^[53]^ Lee et al^[54]^ have clarified that inhibition of the PI3K/Akt signaling pathway can lead to G2/M phase arrest, autophagy, and apoptosis of GC cells. Proteoglycan, an important part of extracellular matrix proteins, can mediate the migration and proliferation of tumor cells.^[55]^ The interleukin-17 signaling pathway can dramatically alter the tumor microenvironment by enhancing the secretion of chemokines and cytokines, thereby promoting tumor occurrence and development.^[56]^ The TNF signaling pathway regulates inflammatory responses in the body. Excessive TNF inflammatory cytokines in the tumor microenvironment can elicit tumor growth and destroy the innate immune response of tumor cells.^[57]^ The HIF-1α signaling is regarded as an initiator of GC progression, which can promote GC cell proliferation and angiogenesis, induce epithelial-mesenchymal transition, and enhance the invasion and metastasis of GC. Consequently, inhibition of the HIF-1α signaling can be a promising strategy to prevent GC cells from infiltration and metastasis.^[58]^

## 5. Conclusion

Through meta-analysis and network pharmacology, our study demonstrated that BZYQD can improve the clinical efficacy of chemotherapy for GC and reduce the incidence of adverse reactions. BZYQD exerts effects on intervening GC mainly by regulating various compounds, targets, and signaling pathways. This study provides a new perspective for further elucidating the efficacy and mechanism of BZYQD in the treatment of GC.

## Acknowledgments

We thank the authors for establishing the relevant database and software. This study has been refined in comparison with the preprint version released on Research Square (https://doi.org/10.21203/rs.3.rs-1098024/v1), and we thank each of the authors who contributed to this study.

## Author contributions

**Data curation:** Ruiping Yang.

**Funding acquisition:** Ruixue Jiang.

**Methodology:** Ruiping Yang, Chunhui Tao, Ruixue Jiang.

**Formal analysis:** Yonggui Wu, Xiaojing Lin.

**Resources:** Chunhui Tao, Ruixue Jiang.

**Investigation:** Ruiping Yang, Yonggui Wu, Chunhui Tao, Xiaojing Lin.

**Supervision:** Ruixue Jiang.

**Visualization:** Ruiping Yang, Yonggui Wu.

**Writing – original draft:** Ruiping Yang.

**Writing – review & editing:** Ruiping Yang.
